# Simplifying carb counting: A randomized controlled study – Feasibility and efficacy of an individualized, simple, patient‐centred carb counting tool

**DOI:** 10.1002/edm2.411

**Published:** 2023-02-07

**Authors:** Shulamit Witkow, Idit F. Liberty, Irina Goloub, Malka Kaminsky, Olga Otto, YonesAbu Rabia, Ilana Harman Boehm, Rachel Golan

**Affiliations:** ^1^ Diabetes Unit, Soroka University Medical Center Beer Sheva Israel; ^2^ Ben‐Gurion University of the Negev Beer Sheva Israel; ^3^ Clalit HMO of the Negev Beer Sheva Israel

**Keywords:** carbohydrate counting, patient instruction, type 1 diabetes

## Abstract

**Introduction:**

The purpose of this study was to introduce and test a simple, individualized carbohydrate counting tool designed for persons with Type 1 Diabetes Mellitus (T1DM) in order to determine whether the tool improved A1C levels for participants with age, education or language barriers.

**Methods:**

In a randomized controlled trial, 85 participants were offered six diabetes instructional sessions free of charge over a six‐month period. Forty‐one received guidance using the regular carbohydrate counting (RCC) method. Forty‐four received guidance using an individualized ‘Simple Carb Counting’ (SCC), involving two customized tables prepared for participants.

**Results:**

The simple, individualized SCC tool for carbohydrate counting was non‐inferior to the standard method of RCC. The SCC tool was more effective among participants aged 40 and older, while no differences were found when comparing participants by education level. Irrespective of intervention group, all participants improved their A1C level (9.9% = 13.2 mmol/L vs 8.6% = 11.1 mmol/L, *p* = .001). A greater improvement in A1C level was seen in newly diagnosed participants (−6.1 vs −0.7, *p* = .005, −3.4 vs 0.9, *p* = .032) in both the RCC and SCC groups. All participants expressed improved emotional level per their PAID5 questionnaires (Problem Areas in Diabetes Scale‐PAID), (10.6 (±5.7) vs 9.5 (±5.7), *p* = .023), with women reporting greater improvement than men.

**Conclusions:**

SCC is a simple, individualized, feasible, low‐tech tool for carbohydrate counting, which promotes and enables accurate insulin dosing in people with T1DM. It was found more effective among participants aged 40 and older. Additional studies are needed to corroborate these findings.

## Graphical abstract

The Scc tool is a simple tool enabling accurate insulin dosing to all diabetes patients treated with basal and bolus insulin. The SCC tool has the potential to apply to all diabetes patients, particular to those who are uncomfortable with the use of advanced technology, or who do not have access to such technology due to age, education or language barriers.

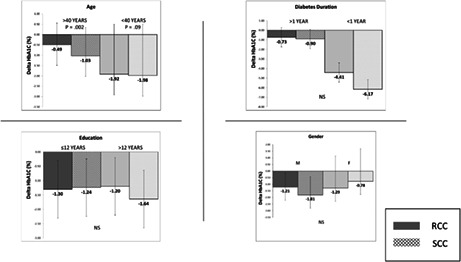

## 1 INTRODUCTION

Achieving glycaemic control in Type 1 diabetes mellitus (T1DM) is vital in order to reduce diabetes‐related complications.^1^, ^2^ Treatment recommendations include multiple daily injections of prandial and basal insulin, or continuous insulin infusion (CSII). As carbohydrates are the major nutrient affecting the post‐prandial response, it is important to educate individuals on matching prandial insulin doses to carbohydrate intake by means of carbohydrate counting (CC). The checking of pre‐meal blood glucose levels and co‐ordination of anticipated physical activity ^3^ are also recommended for optimizing glucose control.

CC takes into account the carbohydrate intake per meal, and enables adjustment of the prandial insulin dosage necessary to achieve individual post‐prandial glucose targets.^4^ To manage CC, the patient has many variables to consider: (i) the personal insulin/carb ratio (I:C) – the amount of insulin the patient needs to overcome a 15‐gram portion of carbohydrates; (ii) the accurate assessment of the amount of carbohydrates consumed; (iii) the insulin sensitivity response (IS), that is, the decrease in blood glucose after injecting one unit of insulin to counter an elevated glucose level; (iv) the individual target glucose level pre‐ and post‐meal; and (v) the level of glucose prior to the meal. This means that in order to normalize post‐prandial glucose levels, individuals need to be highly motivated to maintain wellbeing through on‐going compliant conduct, and to be possessed of high mathematical and literacy skills. Studies^5^ have shown the difficulties of successfully carrying out CC, especially concerning the over‐ and underestimation of carbohydrate content in various foods that can lead to post prandial hypo or hyperglycaemia respectively. In a systematic review^6^ of CC efficacy in managing T1DM, Bell et al^6^ found only partial benefit in achieving glycaemic control through CC, notwithstanding the difficulty of complying with CC instructions. These difficulties are added to other barriers patients with T1DM cope with following dietary recommendations such as, frequent glucose measurements and importantly their feelings about having diabetes.

In recent years, new technologies have been developed to simplify CC.^7^ While various commercial applications and bolus calculators may lead to better glucose control, such options are not available to all populations including lower socioeconomic status, older people and those with lower technological skills. In an attempt to overcome disparities in access and other barriers, the Diabetes Clinic of Soroka University Medical Center (SUMC) developed a simple, easy‐to‐use tool – Simple Carb Counting ‘SCC.’ This tool includes adjustments for those of differing educational backgrounds, cultures and cognitive abilities. It affords persons with diabetes continued enjoyment of their personal eating habits and food preferences through the successful adoption of the SCC method into their daily routines with ease and accuracy.

The aim of this randomized controlled study was to test the feasibility and efficacy of SCC, compared to the regular CC method. Our Hypothesis is that this simple tool will enable all people with T1DM improve diabetes control.

## 2 METHODS

We performed an open‐label randomized controlled trial at the Diabetes Clinic of SUMC, a tertiary 1200‐bed hospital that treats a diverse population, including Bedouin and other Arabs, and Jews from the general, ultra‐Orthodox and Ethiopian sectors. Patients with T1DM were eligible for this study if they were (i) over 18 years old, (ii) treated either with an insulin pump or with multiple daily injections of insulin and (iii) had a hemoglobinA1c (A1C) level equal to, or >8.5. The study excluded pregnant or lactating women, patients with severe renal failure, heart failure or under active treatment for cancer. All participants signed an informed consent statement. The study was approved by the SUMC Helsinki Committee on 8 October 2015, approval number 0320‐15, and is registered at ClinicalTrials.gov, ID NCT04132128, and conducted from November 2015 to July 2017.

Using simple randomization, we assigned participants to one of the two groups, (RCC) Regular carbohydrate counting, or (SCC) simplified individualized tool for carbohydrate counting. The selection process was purely random for every assignment made by Diabetes Clinic staff.

All participants were allowed up to six instructional sessions with a registered dietitian who is also a diabetes care and education specialist during a period of about 6 months. All sessions were free of charge and lasted at least 60 min. During the first session, participants were introduced to the carbohydrate counting method to which they were assigned at randomization. Subsequent sessions were dedicated to reinforcing and practicing their method and changes in insulin dosage parameters were made if needed. All patients treated with insulin pumps were encouraged to use the bolus calculator. The primary end‐point was level of A1C 6 months after the intervention. Additional data parameters were collected including those of demographics, education and duration of diabetes. Weight was measured and blood studies were conducted before and after the intervention to determine baseline A1C and lipid levels. In order to identify depression and diabetes‐related distress, the participants were asked to complete the PAID5 questionnaire (Problem Areas in Diabetes Scale‐PAID)^8^ at baseline and post‐intervention.

### 2.1 Regular carbohydrate counting (CC)

During the instructional sessions, the rationale for carbohydrate counting was explained. Commercial booklets containing a list of the carbohydrate content of foods were provided, and participants were introduced to websites or cellular phone applications designed to assist the public with determining the carbohydrate content of various foods. The participants were taught to calculate the amount of insulin needed using their personal I:C ratio, IS, correction factor, and the glucose target goals prescribed by the Diabetes Clinic team. Participants were encouraged to keep a food diary to assist them with carbohydrate counting.

### 2.2 Simplified individualized carbohydrate counting (SCC)

The SCC tool consisted of two tables written in the participant's native language and adjusted to the participant's specific requirements. Insulin doses were calculated by professional staff using personalized I:C ratios and IS.

*First Table*: The first table, derived from patients' personal IS, listed the number of units that participants needed to administer in order to correct every pre‐meal blood glucose level so as to reach their target glucose.

**FIGURE 1:**
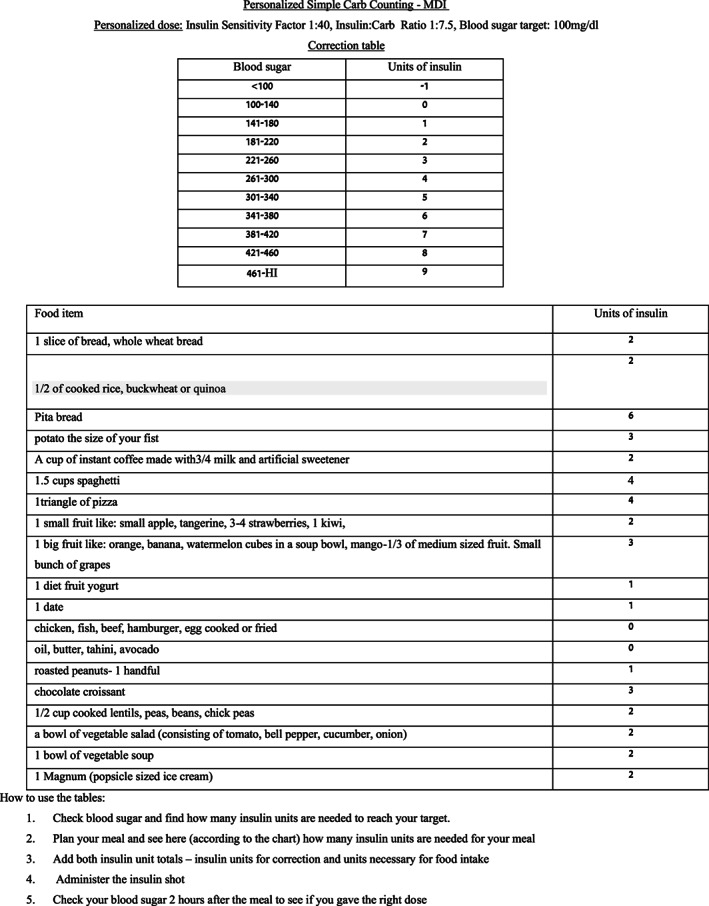
SCC carb counting chart for MDI users.

**FIGURE 2:**
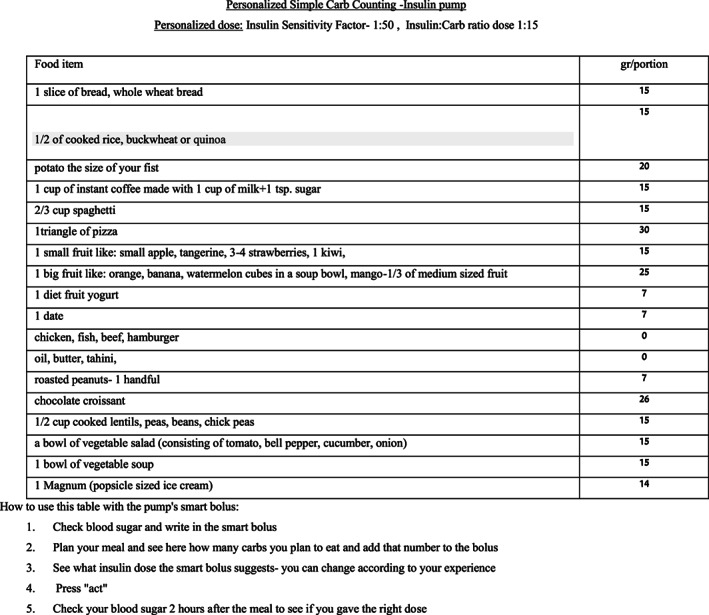
SCC carb counting chart for insulin pump users.

*Second Table*: The second table contained a list of food items derived from participants' personal eating habits, as recorded in their food diaries. The list consisted mainly of the most common foods they regularly consumed, the carbohydrate content of those foods and the number of insulin units needed, as calculated by the personal I:C ratio per usual portion of each food item. High carbohydrate content foods that participants included in their diet were listed, not for healthy nutrition education, but for purposes of facilitating carb counting. Foods that did not contain carbohydrates, such as protein or fat items, were also listed to ensure that the patient realized that these foods contained no carbs. Patients treated with insulin pumps received a personalized table containing the carbohydrates in their food list as grams to accurately calculate the amount of carbs entered to the calculator (Figures 1 and 2).

At each instructional session, participants' tables were reviewed, personal dosing was tested, food items were added to, or deleted from participants' lists as warranted, and the logic of the method was reiterated for the purpose of reinforcing participants' understanding and compliance.

### 2.3 Statistical analysis

On the basis of the expected A1C difference of 0.8% between groups with 90% power providing an SD of 0.4% and a significance level of 0.05% we estimated that the sample size should consist at least 37 participants in each group. We used descriptive statistics to summarize the data, reporting results as means and standard deviations. Categorical variables were summarized as counts and percentages. Paired t‐test was used to examine within changes in A1C between baseline and follow‐up, and Student's *t*‐test was used to examine between differences as to the two intervention groups. We further analysed the data after stratifying the study population by sex, education level (above and below 12 years of school), age (above and below age 40) and duration of diabetes (more or <5 years since diagnosis). *p* values are shown. All analyses were performed with IBM SPSS.

## 3 RESULTS

**FIGURE 3:**
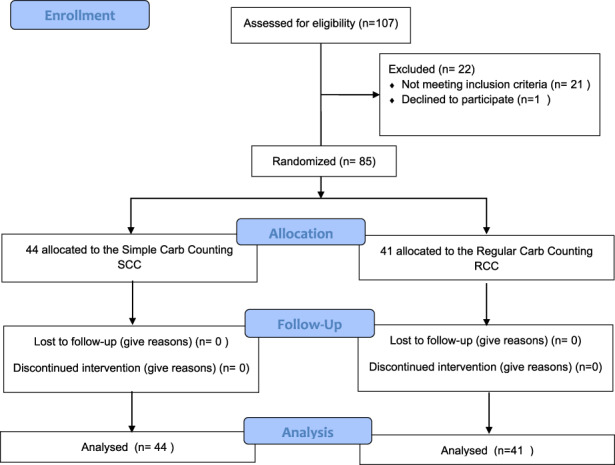
Flow diagram – Trial of standard carb counting versus simple individualized carb counting.

In total, 107 men and women were recruited for the study, of whom 22 were excluded, as shown in Figure 3. Of the 85 people who were deemed eligible, 48.2%, *n* = 41, of participants (23 women, 18 men) were assigned to RCC method group, and 51.8%, *n* = 44, of participants, (17 women, 27 men) to the SCC method group. The mean age of participants was 43.1 (18–74). About 43% of the RCC method group were treated with an insulin pump versus 36% of the SCC group (*p* = .48). All patients monitored their glucose by self‐monitoring of blood glucose (SMBG) (Figure 1 and Table 1).

**TABLE 1:** Baseline characteristics of study population by carb counting method.

Variable	RCC (*n* = 41)	SCC (*n* = 44)	*p* value
Age, Years (±SD)	40.9 (15.5)	45.3 (18)	.18
Women, *N* (%)	23 (56)	17 (39)	.08
Diabetes duration, Years (±SD)	15.2 (12)	16.3 (13.6)	.7
Use of insulin pump *n* (%)	18 (44)	16 (36)	.48
Education above 12 years *n* (%)	17 (42)	21 (48)	.36
BMI kg/m^2^ (±SD)	26.5 (5.0)	25.6 (6.0)	.43
A1C % (±SD)	10.0 (1.9)	9.9 (1.6)	.70
Cholesterol mg/dL (±SD)	178.4 (39.4)	177.8 (42.7)	.95
LDL mg/dL (±SD)	104.8 (31.0)	100.8 (32.2)	.56
HDL mg/dL (±SD)	55.8 (14.2)	50.7 (15.6)	.11
Triglycerides mg/dL (±SD)	106.4 (70.8)	128.3 (57.1)	.12

*Note*: RCC = The regular method of carbohydrate counting (CC); SCC = The individualized simple tool for carbohydrate counting.

Abbreviation: BMI, Body mass index.

The participants were followed for a mean period of 6 months, during which all participants in the study improved their A1C level between baseline and follow‐up (9.9% = 13.2 mmol/L vs 8.6% = 11.1 mmol/L, *p* = .001). Other biomarkers did not change from baseline in either of the groups.

**FIGURE 4:**
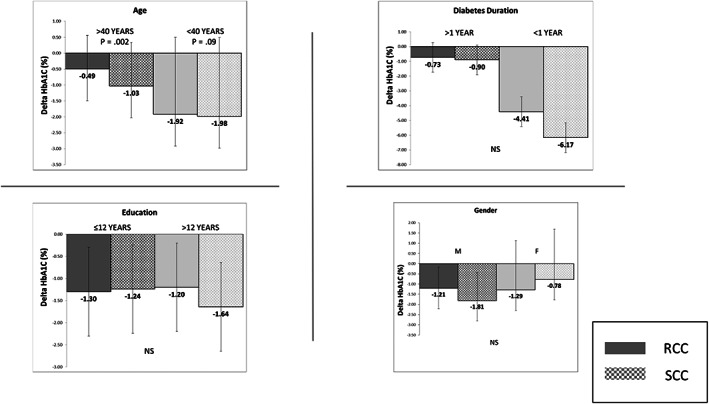
HbA1c results SCC vs RCC.

We stratified the study population by participant age (older and younger than age 40). Among older participants (mean age 55.2 (±9.4)), only those who used the SCC method exhibited significant improvement in A1C level, from baseline (9.6 (±1.3) = 12.7 mmol/L to 8.6 (±1.1) = 11.1 mmol/L, *p* = .002). While those using the RCC method showed some improvement, it did not reach statistical significance (9.7 (±1.3) = 12.9 mmol/L to 9.2 (±1.5) = 12.1 mmol/L, *p* = .09). (Figure 4).

Among younger participants (mean age 29.6 (±6.3)), a significant improvement was found in both the RCC group (10.2 (±2.3) = 13.7 mmol/L to 8.3 (±1.4) = 10.6 mmol/L, *p* = .001) and SCC group (10.3 (±1.9) = 13.8 mmol/L to 8.3 (±1.1) = 10.6 mmol/L, *p* = .002).

We stratified the study population by level of education (above and below 12 school years). Both higher and lower educated participants in the SCC group demonstrated a significant improvement in A1C level. The results for those with above and below 12 school years were −1.4% (±2.0) vs −1.3% (±1.9) respectively, *p* = .7.

We found a greater improvement in A1C levels when we compared participants with more recently diagnosed diabetes (<5 years from diagnosis, *n* = 13) to those whose diabetes was of longer duration (*n* = 72). (−6.1 vs −0.7, span >−3.4 vs 0.9, *p* = .032) in both the RCC and SCC groups.

We measured the degree of patient compliance by the number of instructional sessions attended during the study period. Compliance was fairly good for all participants, with a mean of 4.8 visits (out of the six allowed). No differences were found in compliance between participants in the RCC group and those in the SCC group, but women were more compliant than men, with a mean of 5.3 (1.3) visits, compared to 4.4 (1.9) for the men (*p* = .01). Compliance tended to be higher among participants with more than 12 years of education, compared to those with fewer years of education (4.5 visits (±1.9) vs 5.3 (±1.3), *p* = .08). The degree of compliance correlated with decreased A1C level post‐intervention.

Sixty‐three participants (30 in RCC and 33 in SCC) completed the PAID5 questionnaire at baseline and post‐intervention. All participants reported increased satisfaction, as exhibited by a decreased PAID5 score (10.6 (±5.7) vs 9.5 (±5.7), *p* = .023). Based on the questionnaire responses, there were no differences in diabetes‐related emotional distress between participants in the RCC group and those in the SCC group. Yet, when the investigators stratified the data by sex, they found a significant improvement among women, who reported a decrease in diabetes‐related emotional distress from 11.5 to 9.9, *p* = .04, compared to men, 9.9 to 9.18, *p* = .27 (Table 2).

**TABLE 2:** PAID 5 score results.

	Baseline PAID Score (±SD)	Post‐intervention PAID Score (±SD)	*p*‐value	*p*‐value (SCC vs RCC)
All participants (*N* = 63)	10.6 (5.7)	9.5 (5.7)	.02	
All SCC (*N* = 33)	12.5 (5.4)	11.1 (5.5)	.03	
All RCC (*N* = 30)	9.3 (5.4)	8.42 (5.6)	.09	.69
All women (*N* = 30)	11.5 (5.8)	9.9 (5.6)	.02	
SCC women (*N* = 18)	13.11 (5.5)	11.28 (5.6)	.04	
RCC women (*N* = 12)	9 (5.6)	7.75 (5.1)	.15	.7
All men (*N* = 33)	9.9 (5.7)	9.18 (5.9)	.13	
SCC men (*N* = 12)	10.67 (6.2)	9.83 (6.1)	.23	
RCC men (*N* = 21)	9.48 (5.5)	8.81 (5.9)	.21	.9
Education >12 y (*N* = 31)	8.65 (4.4)	7.87 (4.7)	.12	
SCC education >12 y (*N* = 15)	9.5 (4.9)	8.3 (5.2)	.11	
RCC education >12 y (*N* = 16)	7.9 (3.9)	7.4 (4.4)	.32	.6
Education <12 y (*N* = 32)	12.6 (6.3)	11.1 (6.2)	.02	
SCC education <12 y (*N* = 15)	14.8 (5.5)	13.1 (5.5)	.08	
RCC education <12 y (*N* = 17)	10.6 (6.4)	9.3 (6.5)	.08	.77
Age > 40 (*N* = 33)	9.1 (6.0)	8.1 (5.3)	.07	
SCC age > 40 (*N* = 13)	11.3 (6.6)	9.6 (5.4)	.08	
RCC age > 40 (*N* = 20)	7.75 (5.2)	7.2 (5.2)	.2	.4
Age < 40 (*N* = 30)	12.3 (5.1)	11 (5.9)	.04	
SCC age < 40 (*N* = 17)	12.8 (5.2)	12 (6.1)	.11	
RCC age < 40 (*N* = 13)	11.7 (5.6)	10.3 (5.8)	.11	.9

During the 6 month follow‐up, two patients from the RCC group were hospitalized with diabetic ketoacidosis (DKA). There were no hospitalizations or ER visits due to hypoglycaemia in both groups.

## 4 DISCUSSION

The findings showed that the SCC simple, individualized tool for carbohydrate counting was non‐inferior to the standard method of RCC. The SCC tool was more effective among participants aged 40 and older, while no differences were found when comparing participants above and below 12 school years. However, significant improvement in A1C level was observed in all participants. Participants in both RCC and SCC groups who were diagnosed with diabetes within the previous 5 years exhibited significantly greater improvement in A1C level, compared to participants with diabetes of longer duration.

The SCC method presented in our study was developed in order to overcome difficulties and barriers that the diabetes clinic patients encountered in implementing CC, as described in several studies. Kawamura et al^5^ tested the errors in carbohydrate content estimation among 37 paediatric patients, their parents, and their health care professionals, including physicians and dietitians. In all groups studied, they found overestimation of the carb content in foods with small amounts of carbs, and underestimation in foods with high carb content.

While past experience in CC was important, some foods, such as rice, were hard to estimate even by experienced participants. In the qualitative study of Gürkan et al^9^ investigators interviewed adolescents with diabetes, finding multiple barriers to effective treatment. Among the barriers were patients' negative feelings about having diabetes, as well as personal and environmental barriers. Personal barriers included lack of knowledge about the disease, trouble with glucose measurement, and difficulty following dietary recommendations. These findings were corroborated by Ahola et al^10^ who found that many patients experienced difficulty managing their post‐prandial glucose, and were subject to a high percentage of time in a hyperglycaemic state.

In this study, we present an option to overcome some of the barriers described in the above studies. Through personalization, flexibility, and a departure from a restrictive diet paradigm, SCC affords persons with diabetes an opportunity to continue eating their usual diet, including the customary dishes of their culture, and to go on with their life‐long social dining habits.

The study showed superiority in reaching glycaemic control in participants older than age 40 who used the SCC method, compared to those in the RCC group. Treating older patients with T1DM is complicated by the combined challenges of insulin‐dependent diabetes, age‐related complications, and possible comorbidities, all of which negatively affect the older population's ability to self‐manage diabetes.^11^ The SCC tool presented in the study simplifies the tasks needed for carbohydrate control, and consequently leads to better glycaemic control especially for older age group.

Contrary to these findings, the study demonstrates that SCC was non‐inferior in people with various levels of education. In a cross‐sectional multicentre study of 768 subjects under age 18 with T1DM, Gesuita et al^12^ found that only 28.1% of participants reached target A1C values (<7.5%). A strong correlation was found between higher socio‐economic status (SES), higher level of education and higher ability to follow ordinary CC. Significantly, Gesuita et al. highlighted the need for an accessible tool for non‐privileged populations.

Recently diagnosed participants in both RCC and SCC groups showed the greatest benefit in improving glycaemic control. This may be explained in two possible ways, one psychological and one physiological. When first diagnosed, many patients are highly motivated to do well. In addition, in the early period after diagnosis, sometimes called the ‘honeymoon period,’ the pancreas seems to do better and secretes more insulin, although this phenomenon decreases with time and differs with each patient.

All participants exhibited significant decreases in their PAID5 scores. Studies have shown^13^, ^14^ that people with diabetes suffer from higher levels of psychological distress than does the healthy population. People with T1DM are three times more likely to develop depression than those without diabetes.^15^ Moreover, psychological distress has been shown to be associated with hyperglycaemia, complications and higher mortality rate.^16^, ^17^ Thus, there is a consensus that treating psychological stress and achieving psychological wellbeing ought to be one of the treatment goals of diabetes care.^18^ A study by Zagarins et al^19^ revealed a correlation between improvement of glycaemic control and alleviation of overall psychological stress, but not in depression. Our study corroborates these findings, and underscores the need for on‐going diabetes education, better understanding and treatment of diabetes and promoting a greater sense of self‐efficacy among patients in controlling the disease, as means of improving not only metabolic control, but also mental health.

### 4.1 Limitations

The intervention tool was introduced at a single diabetes clinic in a tertiary teaching hospital with one registered dietitian/diabetes care and education specialist. The method was not tested on paediatric patients, a population that has more difficulties with glycaemic control than others. A larger population of people from the lower socio‐economic and more culturally diverse backgrounds should be studied in order to corroborate the results and establish generalizability across populations.

## 5 CONCLUSIONS AND IMPLICATIONS

In large measure, the research into T1DM treatment is focused on advanced technologies including insulin pumps, continuous glucose monitoring, the artificial pancreas and various applications to support CC and diabetes management. Studies have shown^20^ that although advanced applications are accessible and improve glycaemic control, only a small percentage of the population with T1DM chooses to use them. This may be explained by the human factor, that is, personal expectations, perceptions of the burden of new technologies, user‐friendliness and long‐term cost. The SCC tool tested in this study has the potential to apply to all diabetes patients, and in particular to those who are uncomfortable with the use of advanced technology, or who do not have access to such technology.

In conclusion this study presents a simple, feasible, low‐tech tool that simplifies carbohydrate counting and which promotes and enables accurate insulin dosing in people with T1DM. Additional studies are needed to corroborate these findings.

## AUTHOR CONTRIBUTIONS

**Shulamit Witkow:** Conceptualization (lead); data curation (lead); investigation (equal); methodology (equal); resources (lead). **Idit F Liberty:** Data curation (equal); formal analysis (supporting); investigation (equal); methodology (equal); project administration (equal); supervision (lead); validation (equal); writing – original draft (lead); writing – review and editing (lead). **Irina Goloub:** Data curation (supporting); resources (supporting). **Malka Kaminsky:** Conceptualization (supporting); data curation (equal); investigation (supporting); resources (supporting). **Olga Otto:** Data curation (equal); investigation (supporting). **Yones Abu Rabia:** Data curation (supporting); investigation (supporting); resources (equal). **Ilana Harman Boehm:** Conceptualization (equal); data curation (equal); investigation (equal); methodology (equal); resources (equal). **Rachel Golan:** Formal analysis (lead); writing – review and editing (supporting).

## CONFLICT OF INTEREST STATEMENT

The Authors declare that there are no conflicts of interest.

## Data Availability

Raw data were generated at the diabetes centre of SUMC. Derived data supporting the findings of this study are available from the corresponding author [IFL] on request
